# Unraveling the serotonin saga: from discovery to weight regulation and beyond - a comprehensive scientific review

**DOI:** 10.1186/s13578-023-01091-7

**Published:** 2023-08-07

**Authors:** Kristine Conde, Shuzheng Fang, Yong Xu

**Affiliations:** 1 USDA/ARS Children’s Nutrition Research Center, Department of Pediatrics, Baylor College of Medicine, Houston, USA; 2 College of Art and Sciences, Washington University in St. Louis, St. Louis, MO USA; 3 Department of Molecular and Cellular Biology, Baylor College of Medicine, Houston, USA; 4 Section of Endocrinology, Diabetes, and Metabolism, Department of Medicine, Houston, TX 77030 USA

**Keywords:** Serotonin, 5-HT, Dorsal Raphe Nucleus, Obesity, Appetite, Weight-loss

## Abstract

The prevalence of obesity is rapidly increasing worldwide, while the development of effective obesity therapies lags behind. Although new therapeutic targets to alleviate obesity are identified every day, and drug efficacy is improving, adverse side effects and increased health risks remain serious issues facing the weight-loss industry. Serotonin, also known as 5-HT, has been extensively studied in relation to appetite reduction and weight loss. As a result, dozens of upstream and downstream neural targets of 5-HT have been identified, revealing a multitude of neural circuits involved in mediating the anorexigenic effect of 5-HT. Despite the rise and fall of several 5-HT therapeutics in recent decades, the future of 5-HT as a therapeutic target for weight-loss therapy looks promising. This review focuses on the history of serotonin, the state of current central serotonin research, previous serotonergic therapies, and the future of serotonin for treating individuals with obesity.

## Introduction

The global prevalence of obesity is steadily increasing, with a growing number of affected individuals worldwide. According to the World Health Organization (WHO), obesity is defined as having a body mass index (BMI) greater than 30. In 2016, the WHO estimated that approximately 13% of the world’s adult population had obesity, which is three times higher than the rate in 1975. This estimation accounted for 11% of men and 15% of women [1].

While an imbalance of increased energy intake and reduced physical activity is considered the primary cause of obesity, research in this field has also highlighted genetics, disease, and environmental factors as significant contributors to its development [2–4]. Consequently, diet and exercise, although the most effective weight management therapy, may not yield the same results for everyone. Obesity is associated with an elevated risk of various health conditions such as heart disease, diabetes, depression, cancer, and reproductive impairment, leading to rising healthcare costs. In 2021 alone, the estimated cost of diabetes-related healthcare reached $966 billion, and it is projected to increase to $1,054 billion by 2045 [5].

In recent years, a new injectable glucagon-like peptide 1 agonist called Semaglutide has emerged as a widely prevalent and popular therapy in the weight-loss treatment industry. Clinical trials have demonstrated the effectiveness of Semaglutide in reducing BMI when combined with lifestyle intervention, with once-weekly treatment for adults and adolescents [6, 7]. However, it is important to note that Semaglutide, like any medication, carries associated side effects, including hypoglycemia, gastrointestinal, pancreatic, thyroid, gallbladder, and cardiovascular effects, as well as acute kidney injury, complications related to diabetic retinopathy, and injection-site allergic reactions [8].

While weight-loss therapies have improved over time, serotonergic therapies remain among the leading approaches for effective weight management with new targets continually being discovered. This review focuses on the history of serotonin, the state of current central serotonin research, previous serotonergic therapies, and the future of serotonin for treating individuals with obesity.

### The discovery and diverse roles of serotonin

Serotonin, also known as 5-hydroxytryptamine (5-HT), was initially isolated in 1937 by an Italian pharmacologist named Vittorio Erspamer from the gastric mucosa of a rabbit. At that time, Erspamer referred to it as an “enteramine“[9]. Almost a decade later, in 1948, this enteramine was isolated from bovine serum and given the name “serotonin” due to its vasoconstrictive properties [10]. Serotonin is a highly conserved monoamine. Erspamer successfully identified 5-HT in the gut of various vertebrate animals, including primates, pigeons, frogs, and fish. This finding demonstrated the widespread presence of 5-HT across varied species [11–17].

In mammals, serotonin plays multiple diverse roles. It is involved in regulating various physiological processes, such as gut homeostasis, mood, body temperature, glucose homeostasis, feeding (both homeostatic and hedonic aspects), energy balance, locomotion, migraine, social behavior (including aggression), and circadian rhythm, among others [11–15].

### Serotonin synthesis, storage, and metabolism

5-HT is synthesized from the essential amino acid tryptophan, which is obtained from food. 5-HT is produced in both the peripheral and central nervous systems, and cannot cross the blood-brain barrier [18]. The conversion of tryptophan to 5-hydroxytryptophan is facilitated by the rate-limiting enzyme tryptophan hydroxylase (TPH) [19, 20]. TPH exists in two isoforms, TPH1, found in peripheral serotonin-producing tissues such as the gut, pineal gland, spleen, and thymus, and TPH2 is found in central serotonin-producing neurons like the raphe nuclei [21]. Subsequently, 5-hydroxytryptophan is converted to 5-hydroxytryptamine (5-HT or sertonin) by aromatic l-amino acid decarboxylase [22].

In the brain, 5-HT is stored in vesicles until exocytosis is triggered, leading to its release into the synaptic cleft. In the periphery, the gut is the primary site of 5-HT synthesis; however, platelets will uptake 5-HT from the plasma via the serotonin transporter (SERT), making platelets the fundamental regulators of plasma 5-HT concentration [23]. Platelets store 5-HT in dense granules and release 5-HT into circulation upon stimulation. Once 5-HT is no longer bound to one of its receptors, it is transported back into cells via SERT [24]. Following reuptake, 5-HT is rapidly metabolized by monoamine oxidase into 5-hydroxyindole acetaldehyde, which is further broken down into 5-hydroxindile acetic acid (5-HIAA). The measurement of 5-HIAA, the major metabolite of 5-HT, in urine is a commone and non-invasive method for determining 5-HT levels [25].

### Central serotonin and the raphe nuclei: a complex network

In the brain, the synthesis of 5-HT primarily occurs in the raphe nuclei, which were initially classified into nine nuclei (named B1-B9) in the 1960s [26]. Neurons involved in 5-HT synthesis are present in both the midbrain and hindbrain. The dorsal raphe nucleus (B7 or DRN) located in the midbrain is the main producer of central 5-HT. Interestingly, the DRN has also been implicated as a significant regulator of body weight and feeding [27].

The 5-HT cell groups are numbered in a caudal-to-rostral direction, starting with B1-3 in the medulla, followed by B4-9 in the pons and midbrain. Each number corresponds to a specific nucleus, such as B1 (raphe pallidus), B2 (raphe obscurus), B3 (raphe magnus), B4 (dorsal to prepositus hypoglossi), B5 (raphe pontis), B6 (caudal part of raphe dorsalis), B7 (raphe dorsalis), B8 (centralis), and B9 (supralemniscal nucleus). However, it is worth noting that these nuclei also produce other neurotransmitters, such as Gamma-aminobutyric acid (GABA) and glutamate [28]. Additionally, even within the primary site of central serotonin production, the DRN, there are approximately twice as many non-5-HT neurons as there are neurons that synthesize 5-HT [29, 30].

### Serotonin beyond the blood-brain barrier

Due to its size, serotonin (5-HT) faces difficulty crossing the blood-brain barrier, and therefore its functions in the central nervous system and peripheral tissues are generally considered separate [31]. However, it is important to note that precursors and metabolites of 5-HT may have an easier time crossing the blood-brain barrier [32] and 5-HT can also influence the barrier’s permeability [33–35].

The majority of 5-HT in the body (~ 95%) is produced in the periphery [9, 16, 17, 36]. It is primarily synthesized in enterochromaffin cells of the gut mucosa located in the stomach, and to a lesser extent in the pineal gland and other tissues [37]. In the 1960s, Gershon et al. conducted radioautography experiments using mice and identified that several peripheral tissues, including the adrenal, gastric, thyroid, pancreas, lung, liver, splenic tissues, and blood platelets, take up and store 5-HT [38].

The roles of peripheral 5-HT are diverse, but it primarily regulates gut motility and plays a role in hemodynamics and vasoconstriction [20]. Interestingly, individuals taking Selective Serotonin Reuptake Inhibitor (SSRI) medication commonly prescribed for the treatment of depression, experience gut irregularities such as nausea, constipation, and diarrhea, which can be attributed to the alteration in the gut microbiome caused by the imbalance of 5-HT [39].

### The diverse landscape of 5-HT receptors

There are seven classes of 5-HT receptors, with a current consensus of 14 receptor subtypes in total (Table 1) [40]. The first family of 5-HT receptors consists of five subclasses: 5-HT1A, 1B, 1D, 1E, and 1F. These receptors are G_i_-coupled receptors, and binding of 5-HT to these receptors inhibits adenylate cyclase and reduces cyclic adenosine monophosphate (cAMP) [40, 41]. Additionally, they can indirectly regulate G-protein inwardly rectifying potassium channels, resulting in neuronal hyperpolarization and reduced neuronal activity [40]. Agonism of these receptors leads to anxiolytic and anti-depressant effects.

The 5-HT type 2 receptors consise of three subclasses: 5-HT2A, 2B, and 2C. These are primarily G_q/11_-coupled receptors, and their activation increases inositol phosphate and intracellular calcium concentration. Agonism of 5-HT type 2 receptors exhibits anti-obesity (anorexigenic) and some anti-depressant and anti-physcotic effects [12, 42]. Of note, the 5-HT_2C_ receptor was originally classified as 5-HT_1C_, however it was reclassified due a shared pharmacological profile with the type 2 receptors [43, 44].

The 5-HT type 3 receptor is typically a ligand-gated ion channel, and binding of 5-HT to this receptor rapidly depolarizes neurons through non-selective influx of sodium and calcium [44]. Agonists of the 5-HT 3 receptor remain largely unexplored, but may have anti-psychotic and anti-anxiety properties. Antagonists of the 5-HT 3 receptor are more widely used as an anti-emetic [45].

The 5-HT type 4, 6, and 7 receptors preferentially couple with G_s_ receptors, triggering a second messenger cascade mediated by protein kinase A and increasing cAMP [41, 44]. Agonism of these receptors range from a gastroprokinetic to increase gut motility (5-HT type 4) to anxiolytic and anti-depressant (5-HT type 6) and a potential analgesic (5-HT type 7). Less is known about 5-HT type 5 receptors. There are two subtypes, 5-HT 5A and 5B. They have generally been found to be G_i/o_ coupled, resulting in decreased cAMP. Agonism of 5-HT type 5 receptors may have anxiolytic and anti-depressant effects [41].

**Table 1:** 5-HT Receptor Summary

5-HT Receptor	Receptor Subtypes	Receptor Type	Mechanism of Action	Agonist Effect
5-HT 1	1A, 1B, 1D, 1E, 1F	G_i_-coupled	Decreases cAMP	Anxiolytic and anti-depressant
5-HT 2	2A, 2B, 2C	G_q/11_-coupled	Increases inositol phosphate and intracellular calcium	Anorexigenic and anti-psychotic
5-HT 3		Ligand-gated ion channels	Non-selective influx of sodium and calcium	Anti-psychotic
5-HT 4		G_s_ receptors	Increases cAMP	Gastroprokinetic
5-HT 5	5A, 5B	G_i_-coupled	Decreases cAMP	Anti-migraine and sleep promotion
5-HT 6		G_s_ receptors	Increases cAMP	Anxiolytic and anti-depressant
5-HT 7		G_s_ receptors	Increases cAMP	Anxiolytic and potential analgesic

### Serotonin and appetite: implications for obesity and eating disorders

While 5-HT in the brain only accounts for about 3–5% of the body’s serotonin, it plays a crucial role in regulating appetite [46, 47]. Generally, an increase in 5-HT reduces food intake, while a reduction in 5-HT increases food intake [47, 48]. In fact, after a meal, extracellular 5-HT levels increase in the medial hypothalamus of rats [49]. Notably, 5-HT neurons in the DRN project to the arcuate nucleus of the hypothalamus (ARH), a region well known for its involvement in regulating food intake, energy homeostasis, and body weight [50, 51]. 5-HT agonists provided directly into the brains of rats suppresses food intake and body weight [52]. On the contrary, depleting central 5-HT in rodents leads to a reduction in thermoregulation, a marked decrease in uncoupling protein 1 expression in brown and white adipose tissue, and a sharp increase in blood glucose, free fatty acids and triglycerides [53]. Furthermore, central 5-HT depletion results in increased hyperphagia and body weight gain, eventually leading to obesity [46, 47].

Several investigators have identified neural circuits that may explain the link between psychiatric illness and eating disorders [54, 55]. One of these circuits involves a dysregulated 5-HT system, which is accompanied not only by symptoms of common mood disorders, like depression, but also disordered eating [56]. In fact, mice with mutated or ablated 5-HT_2C_ receptors commonly exhibit hyperphagia, type 2 diabetes, and seizures [57, 58]. For example, patients prescribed atypical antipsychotics, such as olanzapine, often experience noticeable weight gain. Further investigation into olanzapine found that it acts through the 5-HT_2C_ receptor and is suspected to act as an antagonist. Adding Lorcaserin, a high-affinity 5-HT_2C_ receptor agonist mitigates weight gain effect of olanzapine in a mouse model [59]. Karth et al., found that reduced brain 5-HT alters responses to a high-fat diet, such as reduced depression-like behavior and increased anxiety-like behavior, which could explain the correlation between obesity and some mental illnesses [60]. Dieting and malnutrition can reduce the intake of diet-derived tryptophan, leading to reduced serotonin production and availability [61]. In fact, in the 1970s, Breisch and Staller demonstrated that reducing 5-HT synthesis in the brain promotes weight gain and eventually leads to obesity [46, 62]. Furthermore, a reduction or mutation in the 5-HT_2C_ receptor can lead to binge eating behaviors that perpetuate the restricting and binging cycle commonly observed in patients with anorexia nervosa and bulimia [58]. Conversely, overconsumption and obesity can also contribute to a dysregulated 5-HT system. Changes in 5-HT signaling often occur prior to the development of obesity. Mice on a high-fat diet were observed to have an increase in central 5-HT, which may partially contribute to the faster satiating effect of a calorie-dense diet [63]. Additionally, in a study of rats with obesity, 5-HT neurons in the DRN exhibited elevated excitability and had a greater feeding response compared to lean rats [49]. Infusing these rats with 5-HT directly to the ventromedial nucleus of the hypothalamus (VMH), a known feeding-control center of the brain, reduced food intake, but only in lean rats, not obese rats [49, 64]. Furthermore, 5-HT neurons in the DRN (5-HT^DRN^) projecting to the ventral tegmental area (VTA) inhibit hedonic feeding via 5-HT_2C_ receptor and reduced potassium channel currents [51]. Additional studies have also shown that in rats made obese by feeding a high-fat diet exhibit an increase in 5-HT transporter binding in the DRN, ultimately reducing 5-HT availability in the brain [65, 66], which may contribute to an increased feeling of hunger. All of this research demonstrates that obesity can dysregulate the 5-HT pathways in the brain, therefore, making 5-HT an excellent candidate target for anti-obesity treatment. Furthermore, evidence that 5-HT_2C_ receptor agonists have therapeutic potential as a type 2 diabetes medication due to their ability to produce effects on blood glucose and insulin sensitivity independent of weight loss [67, 68].

### Regulation of serotonin, satiation, and the network of feeding control

**Fig. 1:**
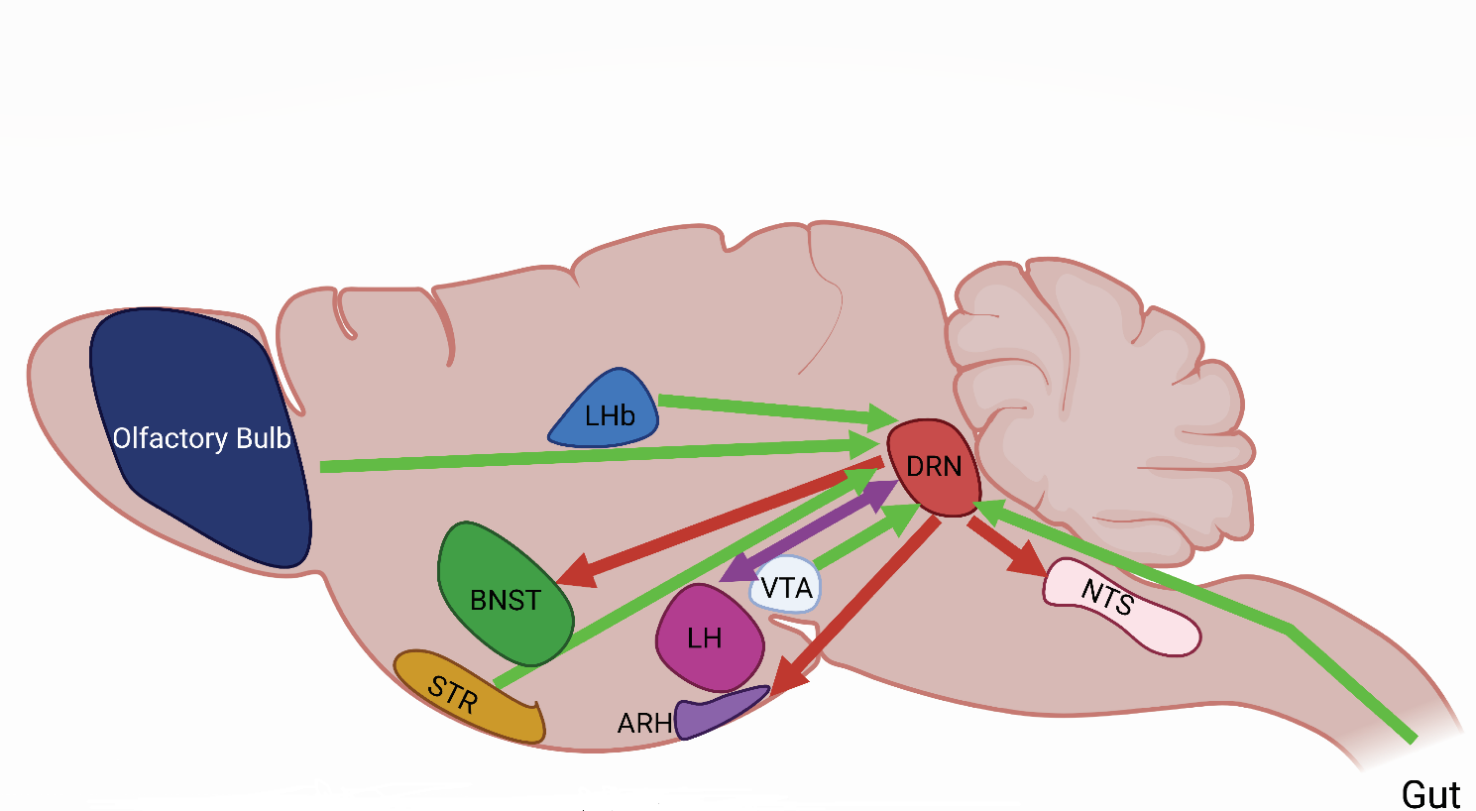
**Projections to and from 5-HT ORN neurons**. Projections to the DRN are illustrated with a green arrow and from the DRN are red. Reciprocal projections are purple. Abbreviations are as follows: ARH, Arcuate Nucleus of the Hypothalamus, BNST, Bed nucleus of the stria terminalis, DRN, Dorsal Raphe Nucleus, LHb, Lateral Habenula, LH, Lateral Hypothalamus, NTS, nucleus tractus solitarius, STR, Striatum.Globus pallidus and substantia nigra are not pictured. Created with BioRender.com

Satiation triggers increases 5-HT activity in both the gut and brain [51, 69]. However, the activation of 5-HT neurons begins long before satiation, likely starting with the smell and anticipation of food. For instance, in *drosophila*, serotonergic neurons respond to the gustatory detection of food, which then signals to downstream insulin-producing cells and enteric neurons (Fig. 1) [70]. This 5-HT activity also communicates with enteric neurons in the gut, promoting gastric motility and initiating the digestion process [70]. It is speculated that this mechanism serves to communicate potential nutrient availability or intake. The signaling of 5-HT continues during the mastication process leading to the increase in 5-HT spike activity [71].

In addition to the 5-HT^DRN^ neurons, dopaminergic neurons in the VTA (DA^VTA^), also receive dense projections from orexin neurons originating from the lateral hypothalamus (LH) and dorsal medial hypothalamus. Orexin neurons, also known as hypocretin neurons, contribute to feeding behavior and body weight homeostasis. In models of orexin deficiency, mice become obese despite consuming fewer calories compared to their lean counterparts [72]. 5-HT^DRN^ neurons receive some of densest projections from orexin neurons (Fig. 1) [73] and are known to project to and inhibit orexin neuronal activity [74]. Additionally, in rats, 5-HT^DRN^ neurons express orexin receptors, OX1R and OX2R [75–77]. In response to orexin, 5-HT^DRN^ neurons exhibit increased inhibitory post-synaptic currents via GABA^DRN^ neurons [78, 79]. Orexin-A and B also inhibit depolarization-stimulated 5-HT release [80]. Moreover, the medial prefrontal cortex has been shown to project to 5-HT^DRN^ neurons, and stimulation of this circuit using optogenetics in rats has a profound effect on depression-like behavior [81]. The DRN also receives input from the lateral habenula which is implicated psychiatric disorders, motivation behavior and depression (Fig. 1) [82, 83]. Viral tracing studies indicate that the DRN is innervated by the striatum, globus pallidus, and substantia nigra [84–87], which regulate autonomic, emotional, aversion and reward-related information. Other inputs to the DRN include the anterior cortex and cerebellar nuclei, which play roles in coordinating sensation, motor control, and cognitive function [88]. Another important player in feeding regulation is the GABA^LH^ neurons that project to the paraventricular hypothalamic nucleus (PVH). Optogenetic stimulation of PVH-projecting GABA^LH^ neurons increases inhibitory post-synaptic current (IPSC) in PVH neurons, leading to increased feeding, while disruption of GABA receptors in the PVH reduced feeding [89]. Notably, GABA^LH^ neurons simultaneously project to 5-HT^DRN^ neurons (Fig. 1) [28], which also increases feeding [51], suggesting a parallel pathway for feeding regulation.

Although this list is not exhaustive, it highlights the regulation of the central serotonergic system by various brain regions and the crucial role played by the 5-HT^DRN^ neurons in controlling and coordinating multiple physiological functions. Notably, while several of these circuits have been studied, few have examined their impact on feeding behavior and body weight, creating an unaddressed gap in knowledge in the field.

### Downstream of 5-HT neurons

Numerous studies have focused on the role of 5-HT^DRN^ neurons projecting to the ARH in inhibiting homeostatic food intake and regulating body weight. Specifically, the innervation of ARH proopiomelanocortin (POMC) and Agouti-related Protein (AgRP) neurons by upstream 5-HT^DRN^ neurons has been extensively studied (Fig. 1) [12, 50, 90, 91]. It has been established that 5-HT_2C_ and 5-HT_1B_ receptors mediate this inhibitory activity [51, 92]. Interestingly, feeding reduces 5-HT responsiveness to GABA inhibitory input, resulting in increased activity of 5-HT neurons [51]. The release of GABA from neurons expressing leptin receptor (LepR) has also been implicated in body weight regulation. Disrupted GABA release from LepR-expressing neurons has been shown to contribute to mild obesity and sensitivity to diet-induced obesity in mice [93]. This mechanism may also play a role in the regulation of 5-HT, but further studies are required to make this determination.

Within the DRN itself, a local circuit has been identified as a regulator of feeding behavior. Neurons expressing vesicular GABA and glutamate transporters (Vgat and VGLUT3) have opposing effects on food consumption, with Vgat^DRN^ neurons increasing and VGLUT3^DRN^ neurons suppressing food intake [27]. Additionally, Vgat^DRN^ neurons inhibit VGLUT3^DRN^ neurons, and 5-HT_1A_ receptor agonist can inhibit TPH2-expressing VGLUT3^DRN^ neurons [27].

The interaction between 5-HT and dopamine activity is also important for feeding regulation. Activation of 5-HT_2C_ receptor stimulates dopamine neural activity and effectively inhibits binge-like eating behavior in mice [94]. Moreover, 5-HT has been shown to control reward processing in the brain through dopamine regulation [95]. The nucleus of the solitary tract (NTS), which expresses 5-HT_2C_ receptors and may receive projections from 5-HT^DRN^ neurons, is involved in feeding behavior (Fig. 1). Activation of POMC neurons via 5-HT_2C_ receptors, in the NTS decreases feeding and mediates acute reduced food intake in response to the selective 5-HT_2C_ receptor agonists, like lorcaserin [96].

Furthermore, selective activation of 5-HT^DRN^ projections to the LH and bed nucleus of the stria terminalis (BNST) triggered by food access and satiety hormones suppresses feeding by increasing extracellular 5-HT (Fig. 1) [97], suggesting redundant circuits mediating the suppression of food intake by 5-HT. In addition, studies have explored the diverse projections of 5-HT^DRN^ neurons to different brain regions, with individual neurons responding to different cues and displaying distinct anatomical subpopulations projecting to reward-related or anxiety-related structures [98–101].

### Exploring serotonergic therapies for weight-loss: progress, challenges, and future directions

Given the extensive communications of the neural 5-HT system with brain regions involved in regulating feeding behavior and body weight homeostasis, it represents a promising target for therapeutic interventions aimed at alleviating obesity. To improve current serotonergic therapies, it is crucial to gain a deeper understanding of their development and current usage [102–104].

SSRIs are commonly used to increase 5-HT availability in the brain by blocking the reuptake of serotonin through SERT. These medications are primarily employed for the treatment of depression. Interestingly, mice deficient in SERT expression develop characteristics such as glucose intolerance, insulin resistance, and obesity, despite reduced food intake [24]. Serotonin reuptake inhibitors, like sibutramine and fluoxetine, as well as monoamine oxidase inhibitors like clorgyline and pargyline, have demonstrated effectiveness in reducing food intake [105–107]. This highlights serotonin as a potential candidate for weight-loss therapies, particularly for individuals who do not respond adequately to diet and exercise alone.

However, it is important to note that many of these serotonin-targeted weight-loss therapies require an intact melanocortin system in order to be effective [108]. Therefore individuals with mutations or deficits in melanocortin receptor expression may not respond favorably to these treatments.

**Fenfluramine and d-fenfluramine**, which are derivatives of amphetamine, elevate extracellular 5-HT levels by disrupting the vesicular storage of 5-HT, leading to increased release. Unlike amphetamines, which increase multiple monoamines like dopamine and norepinephrine, fenfluramine exhibits greater selectivity in increasing 5-HT and has shown lower addictive potential [109, 110]. Fenfluramine was approved as a weight loss treatment in 1973, followed by the approval of dexfenfluramine (d-fen) in 1996. These drugs exert their effects by increasing energy expenditure and reducing body weight, by targeting the lateral hypothalamus [52, 111–114]. In addition, they also target POMC^ARH^ 5-HT_2C_ receptors and downstream melanocortin 4 receptors (Mc4R) in PVH neurons, which are responsible for the appetite-suppressing effects of d-fen [115]. In mouse studies, d-fen dose-dependently reduced the consumption of palatable food, and mice lacking 5-HT_2C_ receptor were less sensitive to these effects [116]. However, chronic treatment with d-fen becomes less effective over time due to a reduction of 5-HT uptake [117–119]. Interestingly, baboons administered repeated fenfluramine did not develop tolerance to its effects on food intake [120]. As a result, these drugs were commonly prescribed in combination with phentermine, an amphetamine, referred to as fen-phen, for short-term weight loss. In human studies, meal microstructure differed between fenfluramine and amphetamine treatments. Both treatments reduce food intake, but fenfluramine specifically reduces the rate of feeding, while amphetamine increases the latency to consume [121, 122]. This study emphasizes the importance of meal microstructure as an often-overlooked aspect of studying appetite in humans. This combination of amphetamine and fenfluramine posed an increased risk for developing pulmonary hypertension and heart disease [123–125]. Consequently, the Food and Drug Administration (FDA) withdrew both fenfluramine and dexfenfluramine from the market in 1997 (Table 2) [42, 115, 125].

**Sibutramine**, which gained approval as an obesity treatment in 1997, replaced fenfluramine, but was subsequently withdrawn in 2010 due to an elevated risk for cardiovascular complications. This drug is a monoamine reuptake inhibitor, primarily used for the treatment of depression. By inhibiting the reuptake of monoamines, such as dopamine, norepinephrine, and serotonin in the central nervous system, Sibutramine increases their concentration [106, 126–128]. While Sibutramine is less effective for depression treatment, it is effective in reducing food intake and increasing energy expenditure, resulting in sustained weight loss [129–131]. However, alongside weight loss, Sibutramine also raises heart rate and blood pressure, thereby increasing the cardiovascular risk for individuals with obesity [132, 133]. Despite an effective reduction in body weight, the associated 16% increase in cardiovascular events prompted its withdrawal (Table 2) [129].

After the withdrawal of Sibutramine, a very promising weight-loss therapeutic called **Lorcaserin**, emerged as a replacement. Lorcaserin is a high-affinity 5-HT_2C_ receptor agonist [134], offering more specific actions compared to the previous serotonin-targeting drugs. The use of a more selective drug aims to minimize off-target side effects associated with non-specific action, such as those observed with Sibutramine. Lorcaserin has shown improvements in glucose tolerance, insulin sensitivity, reduced food intake, and weight loss in obese mouse models, positioning it as a potential candidate for weight-loss therapy [135]. Its mechanism of action involves the downstream Mc4R [68]. Mice lacking Mc4R are not responsive to lorcaserin-induced hypophagia, indicating that melanocortins acting on Mc4R are essential for altering food intake in response to 5-HT_2C_ receptor agonists [14, 134]. Additionally, Lorcaserin has been found to rely on preproglucagon (PPG) neurons in the NTS (PPG^NTS^) to mediate its therapeutic effects on reducing food intake as demonstrated by the lack of response in mice in which PPG^NTS^ neurons are ablated [136].

In human clinical trials, Lorcaserin treatment resulted in modest weight loss and fewer cardiovascular events compared to previous 5-HT-targeted therapies [137, 138], leading to its approval by the FDA as a weight-loss therapeutic [139]. However, rodent toxicology studies revealed abnormal tissue masses in mammary and brain tissues of rats treated with remarkably high doses of Lorcaserin (30 and 100 mg/kg) [140]. At a low dose (3 mg/kg), Lorcaserin was effective in inducing hypophagia and weight loss with minimal side effects [141, 142]. Due to the modest and unsustainable weight loss outcomes and potential carcinogenicity concerns, the FDA ultimately withdrew its approval as a weight-loss aid in 2020 (Table 2) [143–145]. However, Lorcaserin is currently showing potential for treating Dravet Syndrome due to its anti-seizure effects, and clinical trials in this context are ongoing [146].

Further studies have found that the rebound weight gain in individuals taking Lorcaserin is in part attributed to internalization of the 5-HT_2C_ receptor, a common mechanism of G-protein coupled receptors, which results in reduced sensitivity to the effects of Lorcaserin [141]. The reduced sensitivity can potentially be mitigated by adding a β-arrestin inhibitor, although its efficacy in human clinical trials requires further investigation.

**Table 2:** 5-HT Targeted therapies for weight-loss

5-HT-Targeted Therapy	FDA Approval	FDA Withdrawal	Mechanism of Action	Reason for Withdrawal
Fenfluramine	1973	1997	Increase energy expenditure and reduce rate of feeding	Risk for pulmonary hypertension and heart disease
Dexfenfluramine	1996	1997	Reduce consumption of palatable food and increase latency to consume	Risk for pulmonary hypertension and heart disease
Sibutramine	1997	2010	Inhibit reuptake of 5-HT, reduce food intake, and increase energy expenditure	Increased cardiovascular risk
Lorcaserin	2012	2020	5-HT_2C_ receptor agonist, restore glucose tolerance, insulin sensitivity, and reduce food intake	Weight loss unsustainable, potential carcinogenicity

### Conclusions and exploring potential therapeutic avenues: innovative approaches in weight-loss

There are several avenues for further research in the field of 5-HT and weight-loss therapies. Exploring upstream signals to 5-HT^DRN^ neurons, such as GABA and dopamine, could provide additional therapeutic targets for alleviating obesity. While 5-HT_2C_ receptor is the most targeted 5-HT receptor for weight-loss, other receptor subtypes remain largely unexplored in the context of body weight and feeding behavior. The 5-HT_1B_ receptor is one such receptor with exciting potential as a future target for the development of obesity therapeutics. Studies have indicated that co-administration of a 5-HT_1B_ receptor agonist enhances the anorectic effect of 5-HT_2C_ receptor compounds by increasing the number of activated POMC^ARH^ neurons, although not their magnitude, as observed in electrophysiology studies [147]. Recent research further supports the importance of 5-HT_1B_ receptor activation in mediating the hypophagic effects of 5-HT, particularly in AgRP^ARH^ neurons expressing 5-HT_1B_ receptor, which project to the PVH [148].

In the realm of migraine treatment, there have been notable developments with a nasal spray which delivers a highly selective 5-HT_1F_ receptor agonist called lasmiditan. Lasmiditan is the first member of a new drug category of neural acting anti-migraine agents [149]. This therapeutic shows promise as a potential replacement for previous therapies targeting 5-HT_1B/1D_ receptors, which are commonly prescribed for acute migraine attacks. The selective nature, direct nasal delivery, ease of administration for long-term use, and minimal interactions with other 5-HT receptor subtypes makes this type of progressive therapy a potential future approach for weight-loss medications, offering the advantages of targeted efficacy and reduced side effects.

## Data Availability

Not applicable.

## List of Abbreviations

5-HIAA: 5-hydroxindile acetic acid
5-HT: 5-hydroxytryptamine
AgRP: Agouti-related protein
ARH: Arcuate nucleus of the hypothalamus
BMI: Body mass index
BNST: Bed nucleus of the stria terminalis
cAMP: Cyclic adenosine monophosphate
d-fen: Dexfenfluramine
DRN: Dorsal raphe nucleus
FDA: Food and drug administration
GABA: Gamma-aminobutyric acid
IPSC: Inhibitory post-synaptic current
LepR: Leptin receptor
LH: Lateral hypothalamus
Mc4R: Melanocortin 4 receptors
NTS: Nucleus of the solitary tract
OX1R: Orexin receptor 1
OX2R: Orexin receptor 2
POMC: Proopiomelanocortin
PPG: Preproglucagon
PVH: Paraventricular hypothalamic nucleus
SERT: Serotonin transporter
SSRI: Selective serotonin reuptake inhibitor
TPH: Tryptophan hydroxylase
Vgat: Vesicular GABA transporters
VGLUT: Vesicular glutamate transporters
VMH: Ventromedial nucleus of the hypothalamus
VTA: Ventral tegmental area
WHO: World health organization
